# Supplementary figures and images for: Integrated effects of anaerobic soil disinfestation and beneficial microbes in strawberry production
Source: PLoS One. 2025 Dec 2;20(12):e0336999. doi: 10.1371/journal.pone.0336999 (PMC12671810; doi:10.1371/journal.pone.0336999)

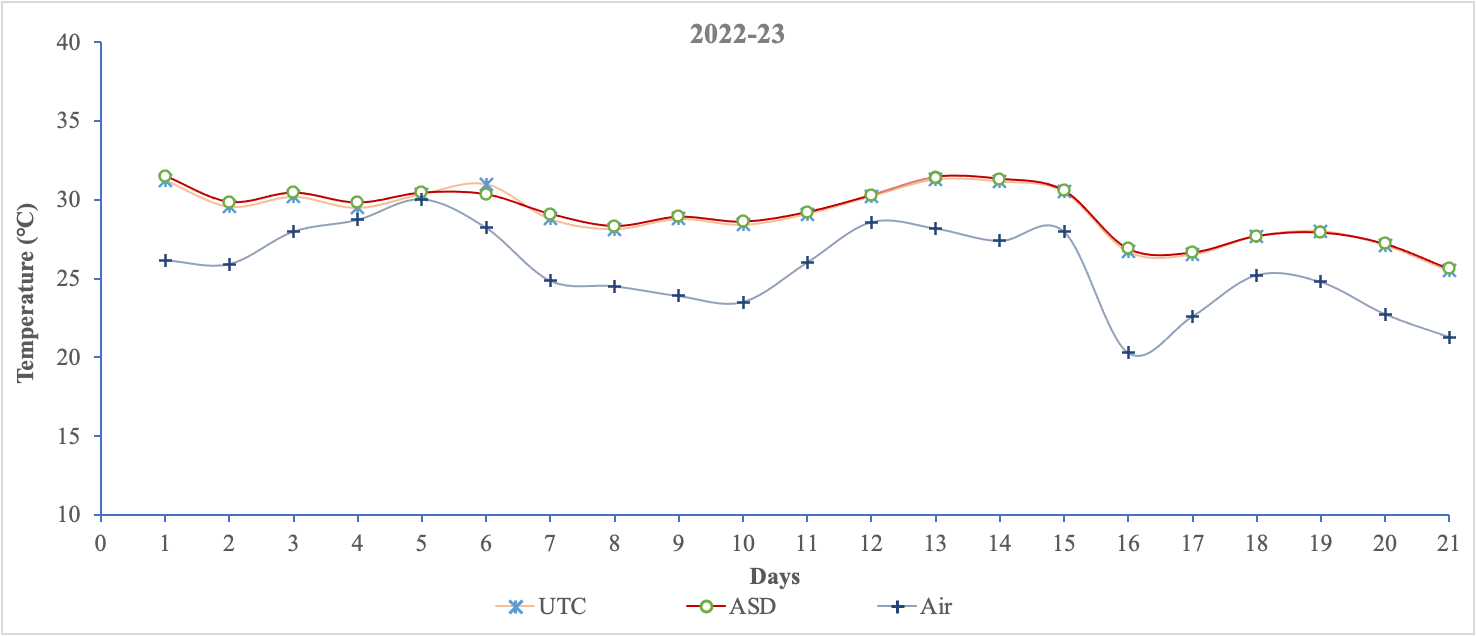


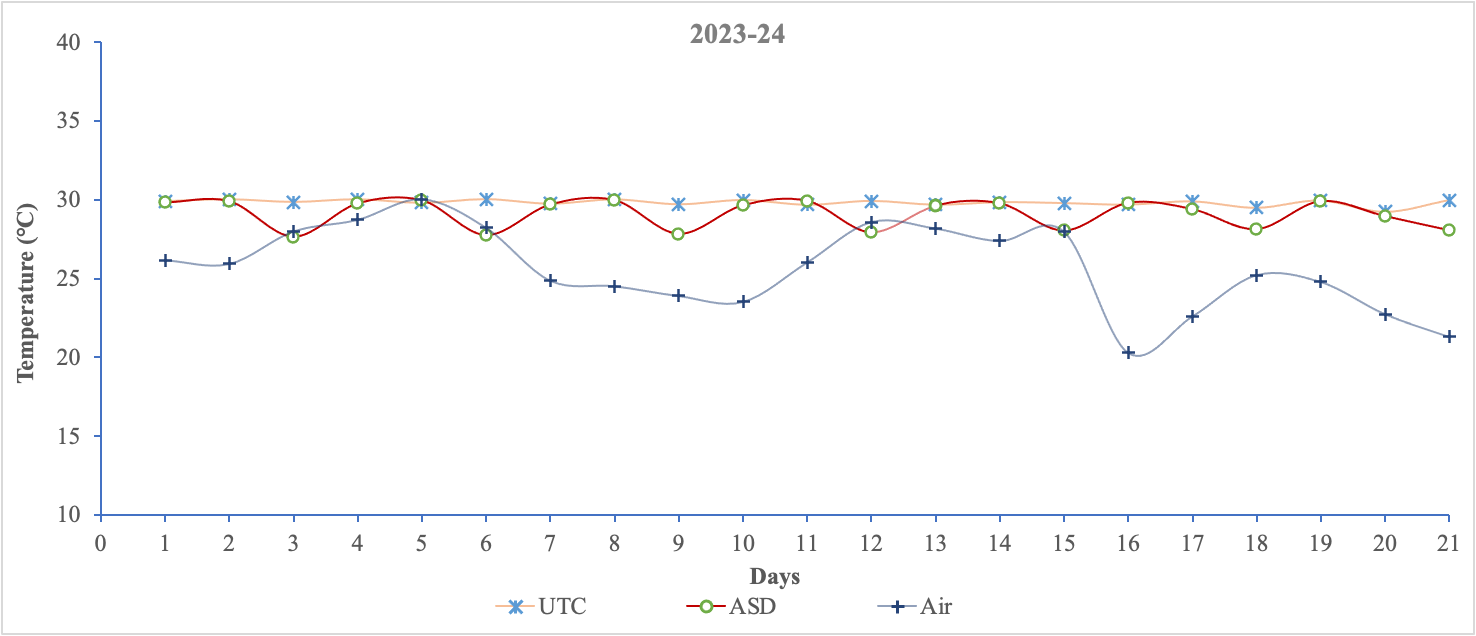

Supplement: S1 Fig — UTC = Untreated; ASD = Anaerobic soil disinfestation, Air = Air temperature. (DOCX) [file pone.0336999.s001.docx]
