# Supplementary material for: Integrated effects of anaerobic soil disinfestation and beneficial microbes in strawberry production
Source: PLoS One. 2025 Dec 2;20(12):e0336999. doi: 10.1371/journal.pone.0336999 (PMC12671810; doi:10.1371/journal.pone.0336999)
Supplement: Attachment — Submitted filename: Response to Reviewers_09_12_25_final.docx [file pone.0336999.s003.docx]

**12 Sept 2025**

**Dear Reviewers**,

Thank you for giving us the opportunity to submit a revised draft of our manuscript titled (**Effects of anaerobic soil disinfestation and beneficial microbes on fruit rot diseases, weed control, and yield in annual hill plasticulture strawberry production**) to (***PLOS ONE***). Our new title is “**Integrated effects of anaerobic soil disinfestation and beneficial microbes in strawberry production”**

We appreciate the time and effort that you and the reviewers have dedicated to providing your valuable feedback on our manuscript. We are grateful to the reviewers for their insightful comments on this manuscript. We have been able to incorporate changes to reflect most of the suggestions provided by the reviewers and those changes are highlighted within the manuscript.

**Here is a point-by-point response to the reviewers’ comments and concerns.**

**Comments from Reviewer #1:**

**We sincerely thank Reviewer #1 for their constructive feedback and positive evaluation of our study. We have addressed all suggested improvements point by point below.**

- **Comment 1**: *Line 37 – if available, you should update with more recent data than 2020*.

**Response:** We have updated the 2020 data with the most recent available figures.

- **Comment *2****: Line 48 – if you start a sentence with a scientific name, then you shouldn’t abbreviate the genus*.

**Response:** We have revised the manuscript to ensure that any sentence beginning with a scientific name includes the full genus name without abbreviation.

- **Comment *3****: Lines 249-253 – it would be good to break this into two sentences.*

**Response:** We have revised Lines 249-253 by splitting the content into two clearer sentences for improved readability.

- **Comment *4****: Line 288 – for your data availability statement to hold true, all data should made publicly available in some way, either in a repository or attached as a supplementary file. I would recommend putting the data in a supplementary file and referencing it appropriately in the text*.

**Response:** Thank you for the suggestion; we have now included all relevant data as a supplementary file and referenced it accordingly in the manuscript to ensure full data availability.

- **Comment *5****:  Line 309 – there is an unnecessary extra space after “during”..*

**Response:** Corrected.

- **Comment *6****: Line 333 – Bacillus should be italicized.*

**Response:** Done.

- **Comment *7****: Lines 471-474 – I think it’s important to acknowledge the need of repeating these experiments at different locations in the future to see if the trends observed in this work still hold true.*

**Response:** We have added a statement emphasizing the importance of conducting these experiments across multiple locations in future studies.

- **Comment *6****:  Lines 700-701 – Move the lines so the are on the same page as table 1..*

**Response:** Moved

- **Comment *7****:  All tables should be moved to the main text of the manuscript. They should be placed following the paragraph in which they were first mentioned.*

**Response:** Done

**Comments from Reviewer #2:**

**Thank you for your positive evaluation of our study’s scope and relevance. We have thoroughly revised the manuscript to address the critical issues raised concerning experimental details, data interpretation, structure, and clarity to enhance its quality and suitability for publication.**

- **Comment 1**: *The title is too long and overly technical. Consider simplifying it while retaining the key elements. Suggested revision: “Integrated effects of anaerobic soil disinfestation and beneficial microbes in strawberry production.”*.

**Response**: Thank you for the helpful suggestion. We have made this change.

- **Comment 2**: *Please include key statistical results (e.g., p-values or percent reductions) to support major claims.’]; Terms like “small farms” and “resource-limited growers” need clarification or citation to avoid generalizations unless supported by data.*

**Response**: Thank you for the valuable feedback. We have now included key statistical results, such as p-values and percent reductions, to substantiate our major findings throughout the manuscript. Additionally, we have clarified the terms “small farms” and “resource-limited growers” with appropriate definitions and citations to ensure accuracy and avoid overgeneralization.

- **Comment 3**: *The introduction is informative but excessively long and somewhat repetitive. The pathogen biology (e.g., AFR, BFR) is discussed in detail, which could be trimmed.*

**Response**: Thanks for the helpful suggestion. We’ve tightened the introduction by removing repetition and condensing the pathogen biology sections (e.g., AFR, BFR) to keep the content focused and easier to read.

- **Comment 4**: *The study’s novelty is not clearly articulated. A more explicit statement of the knowledge gap and the study's unique contribution is needed.*

**Response**: Thank you for highlighting this point. We have revised the introduction to clearly articulate the existing knowledge gap and explicitly state the study’s unique contribution.

- **Comment 5**:  *Some references are outdated (e.g., 2007–2008); replacing them with recent epidemiological or meta-analytical data would strengthen the background.*

**Response**: Thank you for the observation. We have updated outdated references (e.g., 2007–2008) with more recent epidemiological studies and meta-analytical data to strengthen the background and ensure the literature reflects current knowledge.

- **Comment 6**: *The rationale for using a split-split plot design is not adequately explained. Justify why this level of experimental complexity was required.*

**Response**: We appreciate the reviewer’s comment and have revised the manuscript to clarify the rationale for using a split–split plot design.

- **Comment 7**: *Detailed characterization of microbial strains (especially B. velezensis IALR619) is missing. Information on strain origin, viability, concentration, and application rate should be clearly provided.*

**Response**: Thank you for the comment; we have now included detailed information on *B. velezensis* IALR619, including its origin, viability, concentration, and application rate, in the revised manuscript.

- **Comment 8**: t*he carbon source (brewer’s spent grain) used for ASD should be chemically characterized or at least discussed in terms of C:N ratio and prior performance in ASD trials. We cited our previous work to gain more information.*

**Response**: Thank you for the suggestion; we have now included a discussion of the chemical composition of the brewer’s spent grain, including its approximate C:N ratio, and cited relevant studies demonstrating its prior use and effectiveness in ASD applications.

- **Comment 9**: *The statistical approach lacks detail. Indicate the transformations applied, the method for verifying assumptions (normality, homogeneity), and software parameters.*

**Response**: Thank you for the comment; we have now provided a more detailed description of the statistical approach, including the transformations applied, methods for verifying assumptions (normality and homogeneity), and the software parameters used.

- **Comment 10**: *The calculation and use of cumulative redox potential (CuEh) need clarification.*

**Response**: Thank you for your comment; we have now clarified the calculation and use of cumulative redox potential (CuEh) in the revised manuscript, including a detailed explanation of how CuEh was determined over time.

- **Comment 11**: *Explain how CEh was practically applied in the field and link it to the sensor data more clearly.*

**Response**: Thank you for the insightful comment; we have now clarified how cumulative redox potential (CEh) was practically applied in the field by integrating time-series redox measurements from soil sensors, with CEh calculated as the area under the redox potential curve over the ASD treatment period, thereby linking sensor data directly to anaerobic intensity and treatment effectiveness.

- **Comment 12**: *The discussion often summarizes background literature instead of comparing the current results with previous findings. A more focused discussion on how your results support, contradict, or expand upon existing literature is needed.*

**Response**: Thank you for the helpful comment; we have revised the Discussion section to place greater emphasis on how our findings support, contrast with, or extend previous studies, providing a more critical comparison with existing literature rather than summarizing background information.

- **Comment 13**: *• The beneficial effect of ASD on fruit quality is emphasized, but the statistical significance seems modest. Interpret these findings with caution.*

**Response**: Thank you for the observation; we have revised the discussion to interpret the effects of ASD on fruit quality more cautiously,

- **Comment 14**: *The beneficial effect of ASD on fruit quality is emphasized, but the statistical significance seems modest. Interpret these findings with caution.* *Claims about microbial community shifts and suppression mechanisms are speculative, as no microbiome or soil microbial analyses were performed. Please tone down or qualify these statements.*

**Response**: Thank you for the observation; we have revised the discussion to interpret the effects of ASD on fruit quality more cautiously,

- **Comment 15**: *The observed decrease in yield under ASD contradicts the central thesis that ASD enhances production. This discrepancy should be discussed more thoroughly, especially regarding nitrogen immobilization or other nutrient imbalances.*

**Response**: Thank you for the observation; we have revised the discussion to interpret the effects of ASD on fruit yield more cautiously,

- **Comment 16**:  *The effects of B. velezensis inoculation on yield are ambiguous. Some improvements in quality traits are noted, but no significant yield increase is reported. These findings need clearer interpretation.*

**Response**: Thank you for the observation; we have revised the discussion to interpret the effects of ASD on fruit quality more cautiously,

- **Comment 17**:  *The effects of B. velezensis inoculation on yield are ambiguous. Some improvements in quality traits are noted, but no significant yield increase is reported. These findings need clearer interpretation.*

**Response**: Thank you for the observation; we have revised the discussion to interpret the effects of ASD on fruit quality more cautiously,

- **Comment 18**: *Some figures (especially Fig 3.2 and Fig 3.3) are low-resolution and difficult to interpret. Please upload higher-quality versions*.

**Response**: Thank you for pointing this out; we have replaced Figures 3.2 and 3.3 with higher-resolution versions to improve clarity and ensure all elements are easily interpretable.

- **Comment 19**: *Figure legends lack methodological detail. Specify the statistical test used and clarify what error bars represent.*

**Response**: Thank you for the comment; we have revised the figure legends to include methodological details, specifying the statistical tests used and clearly indicating that the error bars represent the standard error of the mean,

- **Comment 20**:  *Table 3.2 is overly complex and crowded. Break it into smaller tables or simplify by focusing on the most relevant results*.

**Response**: Thank you for the suggestion; we have simplified Table 3.2 by focusing on the most relevant results and dividing it into smaller, more readable tables to enhance clarity and accessibility.

- **Comment 21**: *The manuscript is generally well-written but occasionally uses overly complex or repetitive phrasing. Consider simplifying sentence structures for clarity.*

**Response**: We have revised the manuscript to simplify complex or repetitive phrasing and improve overall clarity and readability.

- **Comment 22**: *Phrases such as “our results suggest,” “these findings indicate,” etc., are overused. Improve stylistic variety.*

**Response**: We have now revised the manuscript to reduce repetition of phrases.

- **Comment 23**:  *Rewriting with more natural academic phrasing would improve readability.*

**Response**: Thank you for the helpful comment; we have revised the manuscript to enhance readability by adopting more natural and concise academic phrasing throughout.

- **Comment 24**: *Reference formatting lacks consistency. Some citations include full author lists, others use “et al.” please standardize according to PLOS ONE style.*

**Response**: Thank you for the comment; we have reviewed and standardized all in-text citations and references to ensure consistency with PLOS ONE formatting guidelines, using “et al.” appropriately where required .

**Comment 25**: *DOIs are sometimes duplicated or formatted incorrectly. Review all references for accuracy and consistency.*

**Response**: Thank you for pointing this out; we have thoroughly reviewed all references to correct duplicated or incorrectly formatted DOIs, ensuring accuracy and consistency in accordance with PLOS ONE guidelines.
