# Supplementary material for: Integrated effects of anaerobic soil disinfestation and beneficial microbes in strawberry production
Source: PLoS One. 2025 Dec 2;20(12):e0336999. doi: 10.1371/journal.pone.0336999 (PMC12671810; doi:10.1371/journal.pone.0336999)
Supplement: Attachment — Submitted filename: Response to reviewer comments.docx [file pone.0336999.s004.docx]

**Dear Reviewers**,

Thank you for giving us the opportunity to submit a revised draft of our manuscript titled “**Integrated effects of anaerobic soil disinfestation and beneficial microbes in strawberry production”.** We appreciate the time and effort that you and the reviewers have dedicated to providing your valuable feedback on our manuscript. We are grateful to the reviewers for their insightful comments on this manuscript. We have been able to incorporate changes to reflect most of the suggestions provided by the reviewers and those changes are highlighted within the manuscript.

**Here is a point-by-point response to the reviewers’ comments and concerns.**

**Comments from Reviewer #1:**

We would like to thank Reviewer #1 for their time and consideration of our manuscript.

**Comments from Reviewer #2:**

We sincerely thank Reviewer #2 for their thorough and thoughtful evaluation of our revised manuscript. We truly appreciate the reviewer’s positive remarks acknowledging our efforts to address the major points raised in the previous round. We have carefully considered and addressed all the reviewer’s minor comments as well, which have further improved the precision and quality of our work. We greatly appreciate the reviewer’s constructive feedback and supportive assessment, which have helped us produce a stronger and clearer final version of the manuscript.

- **Comment 1**: *Line 47 (Introduction): The sentence starting with "Strawberries are susceptible..." could use a quick citation for the global impact of AFR and BFR maybe reference [6] earlier to ground it.”*.

**Response**: Thank you for the helpful suggestion. We have added more recent citations to make our point.

- **Comment 2**: *Line 309 (Methods): There's still a minor spacing issue after "during" double-check for any lingering formatting glitches in the final proof.*

**Response**: Thank you for noting this. We have carefully reviewed and corrected the spacing issue after “during,” and verified that no other formatting inconsistencies remain in the revised manuscript.

- **Comment 3**: *Discussion (around Lines 471-474): The added note on replicating at multiple sites is great but consider briefly mentioning potential variables like soil type or climate that could influence outcomes in future work.*

**Response**: We have revised the statement to acknowledge that factors such as soil type and climatic conditions could influence the outcomes of ASD and microbial inoculant applications.

- **Comment 4**: *Tables: Ensure all abbreviations (e.g., AFR, BFR) are defined in the first table footnote for standalone readability.*

**Response**: We thank the reviewer for this observation. All abbreviations have now been defined in the first table footnote to ensure each table is fully understandable on its own.

- **Comment 5**:  *General: A quick proofread for any stray typos e.g., in the abstract, "bacteria (Bacillus velezensis IALR619 and TerraGrow)" could specify if TerraGrow is a product name for clarity, though it's fine as is.*

**Response**: We thank the reviewer for the careful reading and helpful suggestion. We have completed a final proofread to correct any remaining typographical errors. In the abstract, we have clarified that *TerraGrow* is a commercial microbial inoculant product to improve clarity.
